# A Symposium on Scopolamin

**Published:** 1905-12

**Authors:** 


### A Symposium on Scopolamin.

The Dangers of Scopolamine Anesthesia.—Quite recently much has been written in regard to the use of scopolamine as an anesthetic to replace ether and chloroform. The drug is used hypodermically in combination with morphine, and a number of writers are very enthusiastic in regard to its use. Dr. Shoemaker, of Philadelphia, now calls attention to dangers attendant on the use of scopolamine, and in an address which he recently delivered before the Pennsylvania State Medical Society said that he wished to call the attention of physicians to the dangerous toxic properties of the drug. Shoemaker claims that from his experiments he found that even in small quantities, gr. 1/200 to gr. 1/100, its effects are most marked; that it produces in these doses excessive vertigo and extreme dryness of the throat, and that in large doses it may be attended by grave dangers to the patient.—*Medical Age.*

